# Infectious keratoconjunctivitis in semi-domesticated Eurasian tundra reindeer (*Rangifer tarandus tarandus*): microbiological study of clinically affected and unaffected animals with special reference to cervid herpesvirus 2

**DOI:** 10.1186/s12917-018-1338-y

**Published:** 2018-01-16

**Authors:** Javier Sánchez Romano, Torill Mørk, Sauli Laaksonen, Erik Ågren, Ingebjørg H. Nymo, Marianne Sunde, Morten Tryland

**Affiliations:** 1 Department of Arctic and Marine Biology, Arctic Infection Biology, UiT – The Arctic University of Norway, Stakkevollveien 23, 9010 Tromsø, Norway; 2 Norwegian Veterinary Institute, Stakkevollveien 23, 9010 Tromsø, Norway; 3 Department of Veterinary Biosciences, Faculty of Veterinary Medicine, University of Helsinki, Helsinki, Finland; 4 Department of Pathology and Wildlife Diseases, Swedish National Veterinary Institute, 751 89 Uppsala, Sweden; 5 Norwegian Veterinary Institute, Stakkevollveien 23, 9010 Tromsø, Norway; 6 Norwegian Veterinary Institute, Ullevålsveien 68, 0454 Oslo, Norway; 7 UiT – Arctic University of Norway, Arctic Infection Biology, Stakkevollveien 23, 9010 Tromsø, Norway

**Keywords:** Alphaherpesvirus, Gammaherpesvirus, Pestivirus, Bacteria, Eye disease, IKC, Microbiology, Reindeer

## Abstract

**Background:**

Infectious keratoconjunctivitis (IKC) is one of the most common ocular diseases in ruminants worldwide. In addition to keratitis and conjunctivitis, animals with IKC can develop uveitis, corneal ulcer, and in severe cases, blindness. The bacteria *Moraxella* spp. has been described as the primary causative agent of infectious bovine keratoconjunctivitis (IBK) in cattle (*Bos taurus*), while *Chlamydia* spp. and *Mycoplasma conjunctivae* are considered the main causative agents of IKC in sheep (*Ovis aries*). Previous studies indicated cervid herpesvirus 2 (CvHV2) as the primary causative agent of IKC in semi-domesticated reindeer (*Rangifer tarandus tarandus*). The aim of the study was to investigate the presence and prevalence of potential pathogens for IKC in reindeer, and compare the ocular microbiota of animals with IKC, with apparently healthy animals.

**Results:**

Semi-domesticated reindeer (*n* = 341), with (*n* = 108) or without (*n* = 113) ocular clinical signs, or with no information on clinical status (*n* = 120), were sampled in Norway, Sweden and Finland in 2010–2014. Seroprevalence was 37.4% for alphaherpesvirus (95/254), 3.8% for gammaherpesvirus (8/211) and 7.1% for pestivirus (15/211) (ELISA). PCR analyses of conjunctival swab samples revealed a prevalence of 28.5% for CvHV2 (57/200), 11.9% for *Chlamydiaceae* (16/135) and 1.0% for *M. conjunctivae* (2/197). Bacteriological cultivation of 202 conjunctival swab samples revealed bacterial growth from 75.2% of the samples, with *Moraxella* spp. being isolated from 21.6% (11/51) of the animals with and 5.6% (5/84) without ocular clinical signs. A significant association (*p* < 0.001) existed between the presence of clinical signs of IKC and CvHV2 DNA in the affected eyes, an association that was not present for other microorganisms.

**Conclusions:**

These results support the hypothesis that CvHV2 is the primary agent of IKC in semi-domesticated reindeer in Fennoscandia, with *Moraxella bovoculi* being a secondary candidate, since it was isolated in two different outbreaks of IKC. Further studies should be carried out to better understand the infection biology and the pathogenesis of IKC in reindeer.

## Background

Infectious keratoconjunctivitis (IKC) is a severe transmissible ocular disease, which affects many ruminant species worldwide, including reindeer (*Rangifer tarandus*). In cattle (*Bos taurus*), infectious bovine keratoconjunctivitis (IBK) is considered the most important eye disease worldwide [1], and the bacterium *Moraxella bovis* is considered as the main causative agent [2]. However, IBK is regarded as a multifactorial disease, to which also other bacteria, viruses and environmental factors can play a role [3, 4]. In sheep (*Ovis aries*) and goats (*Capra aegagrus hircus*), bacteria from the family *Chlamydiaceae* can be involved in the development of IKC [5]. In sheep in Norway, *Mycoplasma conjunctivae* and possibly *Moraxella (Branhamella) ovis* are considered primary causative agents [6]. IKC has also been described in several wildlife species [7, 8], e.g. chamois (*Rupicapra rupicapra*) [9, 10], alpine ibex (*Capra ibex*) [9] and moose (*Alces alces*) [11]. Many attempts have been made to isolate bacteria from ruminants during outbreaks of IKC, although it is not always obvious if the bacteria isolated have been the responsible pathogen, or if they rather represented opportunistic or environmental bacteria. It has been shown that many different species of bacteria can be cultured from the eyes of apparently healthy ruminants, with no associated clinical signs. Rehbinder and Glatthard [12] indicated that several bacterial species could be cultured from the eyes of 86% of clinically healthy and 90% of diseased reindeer. Barber et al. [13] and Egwu et al. [14] also found this to be true in most clinically unaffected cows (*n* = 261; Scotland, UK) and 97.5% of sheep (*n* = 480; England, UK), respectively.

IKC is a rather common disease in reindeer which usually affects individual animals or small groups, particularly calves and young animals, but the disease may also appear as regular outbreaks, affecting tens or hundreds of animals in a herd, and having major impact on animal welfare and reindeer herding economy [15]. An illustrated questionnaire distributed to reindeer herders in Norway and Sweden revealed that 55.0% of the responding herders (35/63) had observed clinical signs similar to IKC the previous year (2010) [16]. This disease has been described in reindeer for more than 100 years [17]. IKC in reindeer is considered a multi-factorial disease. Many types of bacteria have been isolated from reindeer with IKC, such as *Moraxella bovoculi*, *Moraxella ovis*, *Escherichia coli*, *Listeria monocytogenes* or *Staphylococcus* sp. [18–20], which all may play a role in the development of the disease. However, from an outbreak of IKC in reindeer it was concluded that Cervid herpesvirus 2 (CvHV2) was the causative and transmissible agent, accompanied by secondary opportunistic bacterial infections [15] and it has recently been shown experimentally that CvHV2, alone and in combination with Moraxella bovoculi, was able to cause clinical sympotms characteristic for IKC in reindeer [21].

Other viruses may be relevant in ocular diseases, such as gammaherpesvirus and pestivirus. Antibodies against a virus from the subfamily *Gammaherpesvirinae* were detected in semi-domesticated reindeer in Finnmark County, Norway, with a prevalence of 3.5% in 2013 [22], and the presence of a novel gammaherpesvirus was also found to be circulating among semi-domesticated reindeer in Norway [23]. Other viruses in this subfamily are associated with malignant catarrhal fever (MCF) in different wild and domestic ruminant species, which may cause keratoconjunctivitis, with ocular discharge and panophthalmitis, as seen in cattle [24, 25], or ocular discharge, keratitis and conjunctival hyphema, as observed in American Bison (*Bison bison*) [26]. Serological screenings have demonstrated that pestivirus infections are enzootic in semi-domesticated reindeer in Norway [27, 28] and Sweden [29] and the susceptibility of these animals to bovine viral diarrhea virus infection (BVDV; family *Flaviviridae*, genus *Pestivirus*) has been experimentally demonstrated [30]. A pestivirus (V60-Krefeld; Reindeer-1), genetically related to border disease virus (BDV), was isolated from a captive reindeer in Duisburg Zoo (Germany) [31]. However, pestivirus has not yet been isolated from wild or semi-domesticated reindeer [32]. Some ocular signs, including ocular discharge, conjunctivitis and hemorrhages in the sclera and palpebral conjunctiva, have been reported in cattle infected with BVDV [33], but the possible association between pestivirus infection and IKC in reindeer is not known.

Environmental factors, such as stress, dust, or UV light have also been proposed to contribute to the development of the disease [15, 34–36]. According to the questionnaire survey, IKC in reindeer is most often seen during September to November in connection with the collection, transport and handling of reindeer, which also coincides with the period when animals are observed more carefully and closely, and also when animals are more stressed [16].

## Methods

The aim of the study was to investigate the presence and prevalence of potential pathogens for IKC in reindeer, and to compare the ocular microbiota of animals with IKC, to that of apparently healthy animals.

### Sampling of reindeer

**Fig. 1:**
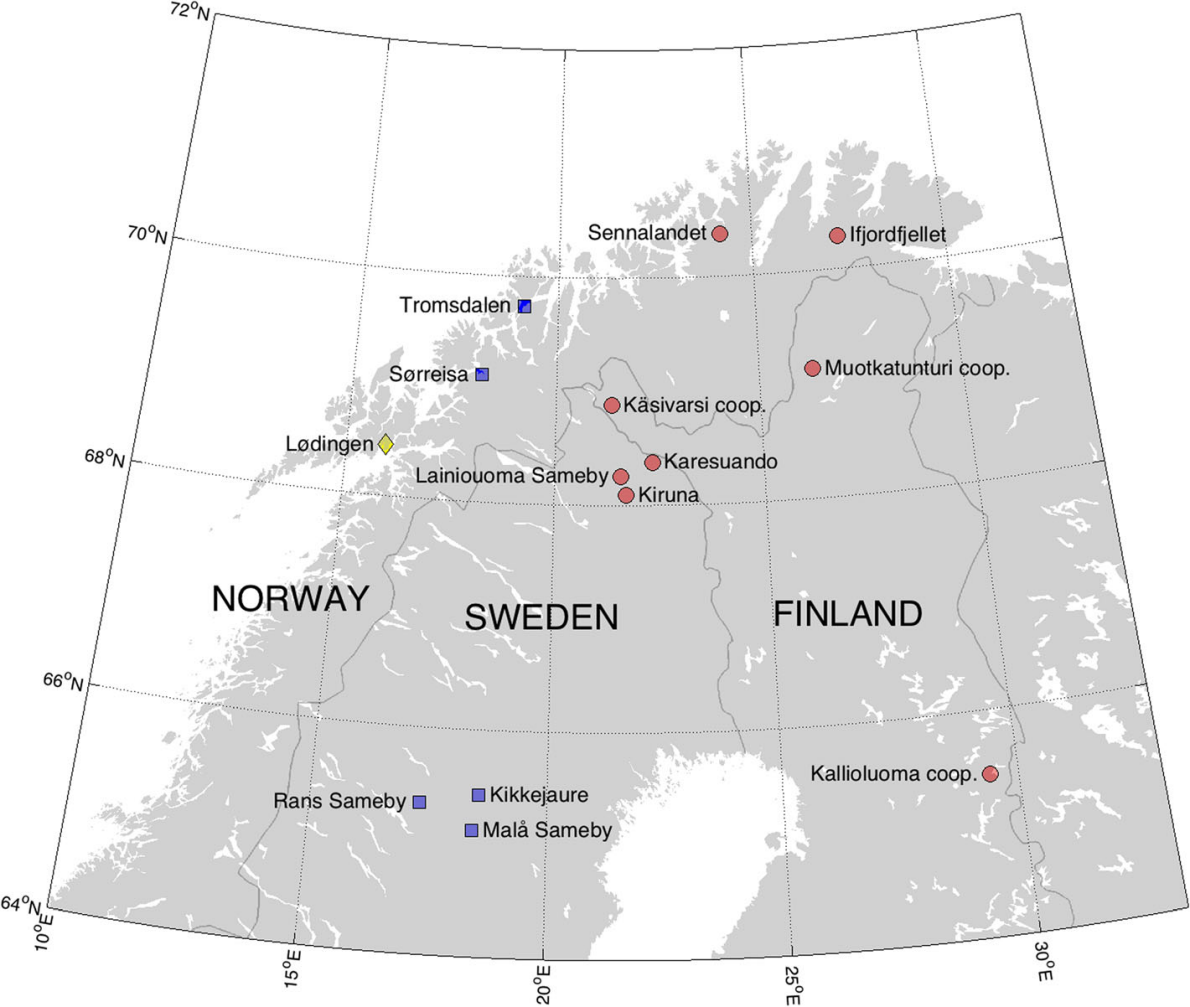
Distribution of sampling sites of semi-domesticated reindeer (*n* = 332) with names of the administrative units (siida, sameby and cooperatives, in Norway, Sweden and Finland, respectively), indicating sampling of live animals (blue squares), slaughtered animals (red circles) or both live and slaughtered animals (yellow diamond)

Semi-domesticated reindeer (*n* = 341) were sampled in Norway (*n* = 171), Sweden (*n* = 139) and Finland (*n* = 31) (Fig. 1) in the period 2010–2014, either during herd gatherings (live animals, *n* = 143) or from slaughterhouses (dead animals, *n* = 198).

In both cases, reindeer with clinical signs of IKC were prioritized for sampling when observed. In total, 108 animals had clinical signs related to IKC, 113 did not show such signs, and for 120 animals, information on clinical signs was not available (Table 1).

**Table 1:** Semi-domesticated reindeer sampled in 2010–2014 in Norway, Sweden and Finland

		Reindeer (all ages) sampled^1^					Reindeer calves (≤1 year old) sampled					Reindeer adults (>1 year old) sampled				
		Ocular clinical signs ^b^					Ocular clinical signs ^b^					Ocular clinical signs ^b^				
Country	Location	Total	0	1	2	NR	Total	0	1	2	NR	Total	0	1	2	NR
Norway	Ifjordfjellet	28	-	2	-	26	9	-	1	-	8	12	-	1	-	11
	Lødingen	49	20	-	-	29	-	-	-	-	-	20	20	-	-	-
	Sennalandet	23	-	2	-	21	6	-	-	-	6	12	-	-	-	12
	Sørreisa	35	9	10	16	-	26	5	8	13	-	9	4	2	3	-
	Tromsdalen	36	30	6	-	-	27	27	-	-	-	9	3	6	-	-
Sweden	Karesuando	34	16	7	11	-	34	16	7	11	-	-	-	-	-	-
	Kiruna	20	8	12	-	-	20	8	12	-	-	-	-	-	-	-
	Kikkejaure	32	2	1	5	24	-	-	-	-	-	-	-	-	-	-
	Lainiouoma Sameby	33	-	21	12	-	27	-	20	7	-	6	-	1	5	-
	Malå Sameby	5	-	-	-	5	2	-	-	-	2	3	-	-	-	3
	Rans Sameby	15	-	-	-	15	10	-	-	-	10	4	-	-	-	4
Finland	Kallioluoma cooperation	10	9	-	1	-	10	9	-	1	-	-	-	-	-	-
	Käsivarsi cooperation	11	9	1	1	-	11	9	1	1	-	-	-	-	-	-
	Muotkatunturi cooperation	10	10	-	-	-	10	10	-	-	-	-	-	-	-	-
		341	113	62	46	120	192	84	49	33	26	75	27	10	8	30

^a^The number of reindeer (all ages) sampled includes animals from which the age information was unavailable, together with the number of calves and adults.

^b^The severity of the ocular disease was scored with 0 for asymptomatic animals, 1 for animals with increased lacrimation and/or mild conjunctivitis or 2 for animals with moderate to severe clinical signs of IKC. Animals from which information was not registered were placed in column NR

The age of the animals was determined by reindeer owners. Animals born in the previous calving season and therefore under 1 year of age were registered as calves (*n* = 192). Animals born in previous calving seasons were registered as adults (*n* = 75), and for 74 animals, age-class information was not available.

For the animals with clinical information available, severity of the ocular disease was scored from 0 to 2, with 0 for asymptomatic animals, 1 for animals with increased lacrimation and/or mild conjunctivitis or 2 for animals with moderate to severe clinical signs of IKC, including corneal and periorbital oedema, conjunctivitis, keratitis, pus with or without blood in the eye or its surrounding area, corneal ulcus or collapse and fibrosis of the eye.

Blood samples were collected from the jugular vein in blood tubes with and without K2EDTA (BD Vacutainer®; BD, Plymouth, UK), using a venoject needle (Terumo, Leuven, Belgium) for live animals, or by collecting blood directly into open tubes during bleeding of slaughtered animals. Tubes were centrifuged for 10 min at 3.000 g to prepare serum or plasma and were then stored at −20 °C until analysis.

Swab samples for virology (Applimed SA, Châtel-St-Denis, Switzerland) were inserted into the conjunctival fornix, rubbed gently against the conjunctival mucosa and placed in sterile cryotubes with 800 μl of Eagle’s Minimum Essential Medium (EMEM) with antibiotics (final concentrations of 100 IU/ml of penicillin, 100 μg/ml of streptomycin, 50 μg/ml of gentamicin and 2.5 μg/ml amphotericin B) and stored at −80 °C until analysis.

Swab samples for bacteriology were obtained from the conjunctival fornix as described for the virology swabs and placed in Amies transport medium with charcoal (Transwab® Amies Charcoal Transport; MWE, Wiltshire, England), transported unfrozen to the laboratory and cultured within 2–9 days after sampling, depending on transport time to the laboratory.

When the reindeer was sampled both for virology and bacteriology, the bacteriology swab was obtained immediately after the virology swab and from the same eye.

### Serology

Serum samples were tested for the presence of antibodies against alphaherpesvirus with a commercial BoHV1 blocking enzyme-linked immunosorbent assay (bELISA) kit (LSI, Lissieu, France) previously validated for the testing of reindeer serum samples for CvHV2 specific antibodies [37]. Positive and negative controls for cattle provided in the bELISA kit were included on each plate.

A direct competition ELISA (cELISA) for the detection of antibodies against the MCFV group [38] was performed to detect the presence of gammaherpesvirus antibodies in reindeer serum as previously described [22].

A serological screening was also carried out for the detection of antibodies against pestivirus using a commercial bELISA kit for the detection of BVDV antibodies in cattle (SERELISA™ BVD p80 Ab Mono blocking; Synbiotics Europe SAS, Lyon, France), which has been previously evaluated for testing reindeer serum samples [39].

### Polymerase chain reaction analysis

DNA was extracted from swab samples with a QIAamp DNA MiniKit (Qiagen, Hilden, Germany) following the manufacturer’s instructions. Different subsets of reindeer samples were analyzed for the presence of DNA specific to CvHV2 (*n* = 200), *Chlamydiaceae* (*n* = 135) or *M. conjunctivae* (*n* = 197) depending on the availability of DNA extracted from the swab samples.

A nested pan-alphaherpesvirus PCR targeting the UL27 gene encoding glycoprotein B (gB) of CvHV2 was performed as described by Ros and Bèlak [40] for CvHV2 (strain Salla82, Finland; Ek-Kommonen et al. [41], initially named rangiferine herpesvirus 1), in a subset of samples (n = 200). DNA extracted from purified CvHV2 was used as positive control and diethylpryocarbonate (DEPC) water was used as negative control.

One hundred and thirty-five (*n* = 135) samples were analyzed (National Veterinary Institute, Sweden) by a TaqMan real-time PCR specific for members of the family *Chlamydiaceae*, targeting the 23S rRNA operon [42]. The cut-off value was set at C_t_ > 38, so every sample with a threshold cycle (C_t_) below that was considered positive for the presence of *Chlamydiaceae* DNA.

A specific PCR assay based on unique sequences of the *LppS* gene [43] was used for detection of *Mycoplasma* spp. in a subset of samples (*n* = 197). DNA purified from *M. conjunctivae* strain HRC/581 originating from sheep (ATCC 25834; NCTC 10147) was used as positive control [44], while DEPC water was used as negative control.

Amplified DNA products were separated by agarose gel (1.0%) electrophoresis and stained with ethidium bromide. Amplicons similar to the expected size (139 bp for *M. conjunctivae* and 294 bp for CvHV2) were purified and sequenced. Consensus amplicon sequences were assembled with the Chromas pro software (version 1.7.7, Technelysium Pty Ltd., South Brisbane, QLD, Australia) and blasted in GenBank (NCBI, USA) for confirmation and comparison to available matching sequences of CvHV2 or *M. conjunctivae.*

### Bacteriological investigation

Swab samples from the conjunctiva of the most affected eye in animals with clinical signs of IKC, and one of the eyes when sampling apparently healthy animals were cultivated on two 5.0% sheep blood agar plates, incubated aerobically and anaerobically, and on one lactose-saccharose-bromothymol blue agar plate (equal to MacConkey agar) incubated aerobically. All plates were incubated at 37 °C and inspected after 24 and 48 h. Bacterial growth was categorized as rich, moderate, or poor. Dominant colonies or colonies suspected as relevant, were subcultured for purity and characterized (morphology, Gram staining, catalase, oxidase). API® strips (bioMérieux, Marcy l’Etoile, France) were used for bacterial identification. If identification to species level was not successful, isolates were characterized by 16S ribosomal RNA (16S rRNA) gene sequencing. The proximal part of the 16S rRNA gene was amplified and sequenced using the MicroSeq® 500 16S rRNA Bacterial Identification Kits (Applied Biosystems, Foster City, CA, USA). The sequencing reactions were run on a capillary sequencer 3130xl Genetic Analyzer (Applied Biosystems Life Technologies, Thermo Fisher Scientific Inc., Waltham, MA). Sequences were analyzed using the CLC bio Combined Workbench (CLC bio, QIAGEN, Aarhus, Denmark) or the BioEdit program (http://www.mbio.ncsu.edu/bioedit/bioedit.html) and homology search was performed using NCBI GeneBlast2 program.

### Statistical analysis

All statistical analysis was performed using SYSTAT 13 for Windows, using Fisher’s exact test. Differences were considered significant when *p* < 0.05.

## Results

### Serology

**Fig. 2:**
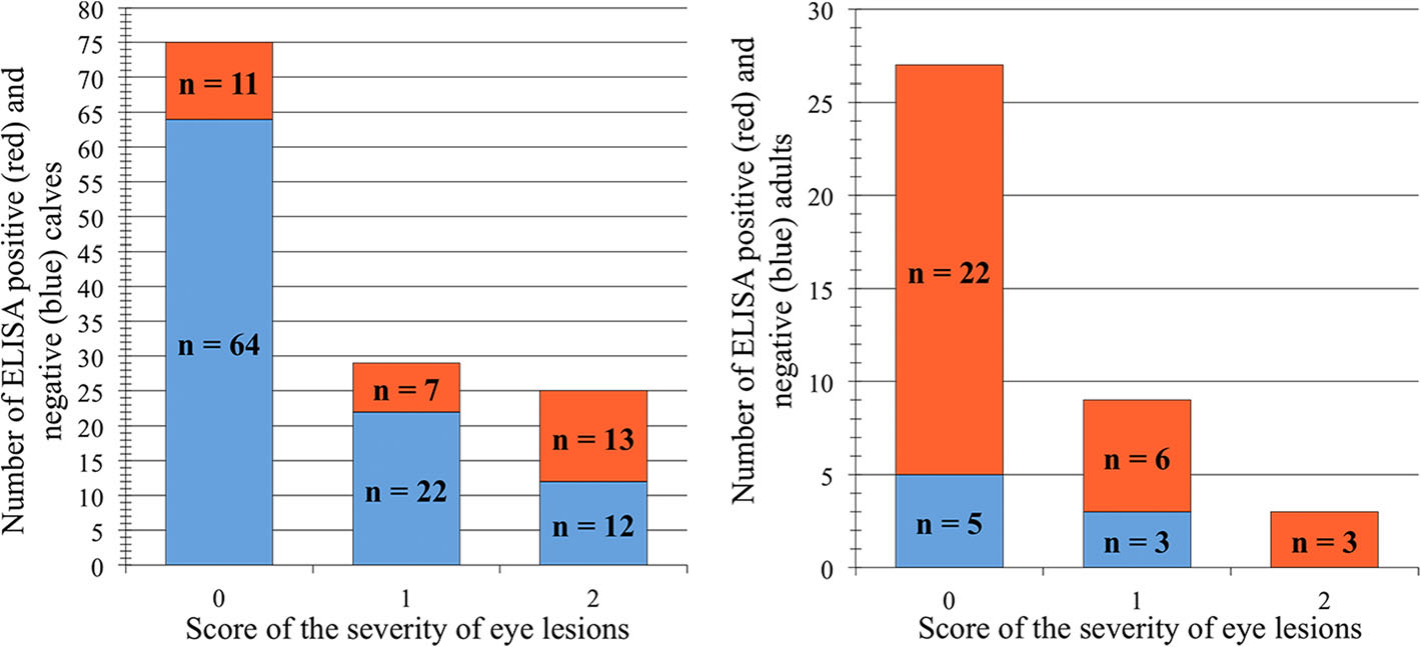
Alphaherpesvirus seroprevalence among reindeer without eye lesions (0), with mild (1), or moderate/severe (2) eye lesions presented as seronegative (blue) and seropositive (red) comparing calves (left) and adults (right)

Seroprevalence of antibodies against alphaherpesvirus was 79.5% (31/39) in adults and 24% (31/129) in calves. For calves, the seroprevalence increased with increasing severity of clinical signs, while for adults, no such difference was present and the seroprevalence was 66.7–100% regardless of the clinical score (Figs. 2 & 4).

There was a statistically significant and positive association between the presence of clinical signs of IKC and the presence of antibodies against alphaherpesvirus in calves (*p* = 0.006). No association was found for adults (*p* = 0.682) or for the whole sample set when age class was not specified (*p* = 0.143).

Eight out of 211 animals (3.8%) were seropositive for the presence of antibodies against gammaherpesvirus. Among animals with known clinical status (*n* = 140), it was not possible to establish an association between the presence of antibodies against gammaherpesvirus and the presence of clinical signs of IKC (*p* = 0.257), a lack of association that persisted also when addressing calves (*p* = 0.394) or adults (*p* = 0.312) separately.

The presence of antibodies against pestivirus could not be associated with the presence of clinical signs of IKC, either when analyzing calves and adults together (*n* = 140; *p* = 0.761), or among just calves (*p* = 1.000). Since all seropositive animals were calves no such calculation was possible for adults as a separate category. If animals without known clinical information were included (*n* = 211), 15 reindeer had antibodies against pestivirus (7.1%). Six additional animals were classified as doubtful in the ELISA, retested with the same results, and subsequently excluded from our statistical analysis.

In total, 202 animals were screened for the presence of antibodies against all the three viruses (Table 2). Among them, ten animals had antibodies against pestivirus and alphaherpesvirus, while four had antibodies against gamma- and alphaherpesvirus. None of the animals were seropositive for both pestivirus and gammaherpesvirus, or for all the three viruses. Among the pestivirus seropositive animals, 66.7% (10/15) were also seropositive to alphaherpesvirus, with a statistical significant association between the presence of antibodies against alphaherpesvirus and pestivirus (*p* = 0.003). This association did not exist in any of the other combinations of antibodies.

**Table 2:** Semi-domesticated reindeer screened by ELISA (*n* = 202) for antibodies against alphaherpes-, gammaherpes- and pestivirus

	Ocular clinical signs ^a^			
	0	1	2	Not registered
Alphaherpesvirus	29 / 89 (32.6)	7 / 24 (29.2)	16 / 27 (59.3)	10 / 62 (16.1)
Gammaherpesvirus	3 / 89 (3.4)	2 / 24 (8.3)	2 / 27 (7.4)	1 / 62 (1.6)
Pestivirus	7 / 86^b^ (8.1)	4 / 24 (16.7)	1 / 27 (3.7)	3 / 59^b^ (5.1)
Gammaherpesvirus and Alphaherpesvirus	1 / 3	1 / 2	2 / 2	0 / 1
Pestivirus and Alphaherpesvirus	6 / 7	4 / 4	0 / 1	0 / 3

Results are presented as positive/screened (%). No individuals were seropositive for both gammaherpes- and pestivirus, nor for the combination of all the three viruses

^a^The severity of the ocular disease was scored with 0 for asymptomatic animals, 1 for animals with increased lacrimation and/or mild conjunctivitis or 2 for animals with moderate to severe clinical signs of IKC

^b^Three samples were considered doubtful and run twice with the same result and therefore excluded from the analysis

### Cervid herpesvirus 2 detection

**Fig. 3:**
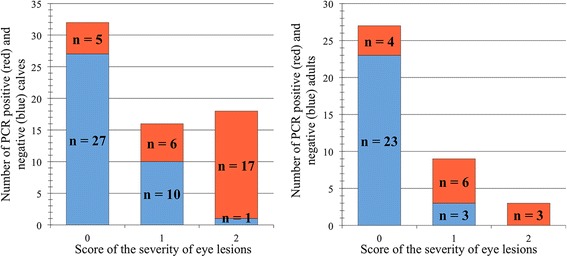
Alphaherpesvirus PCR results (eye swab) among reindeer without eye lesions (0), with mild (1), or moderate/severe (2) eye lesions presented as PCR negative (blue) and PCR positive (red) comparing calves (left) and adults (right)

**Fig. 4:**
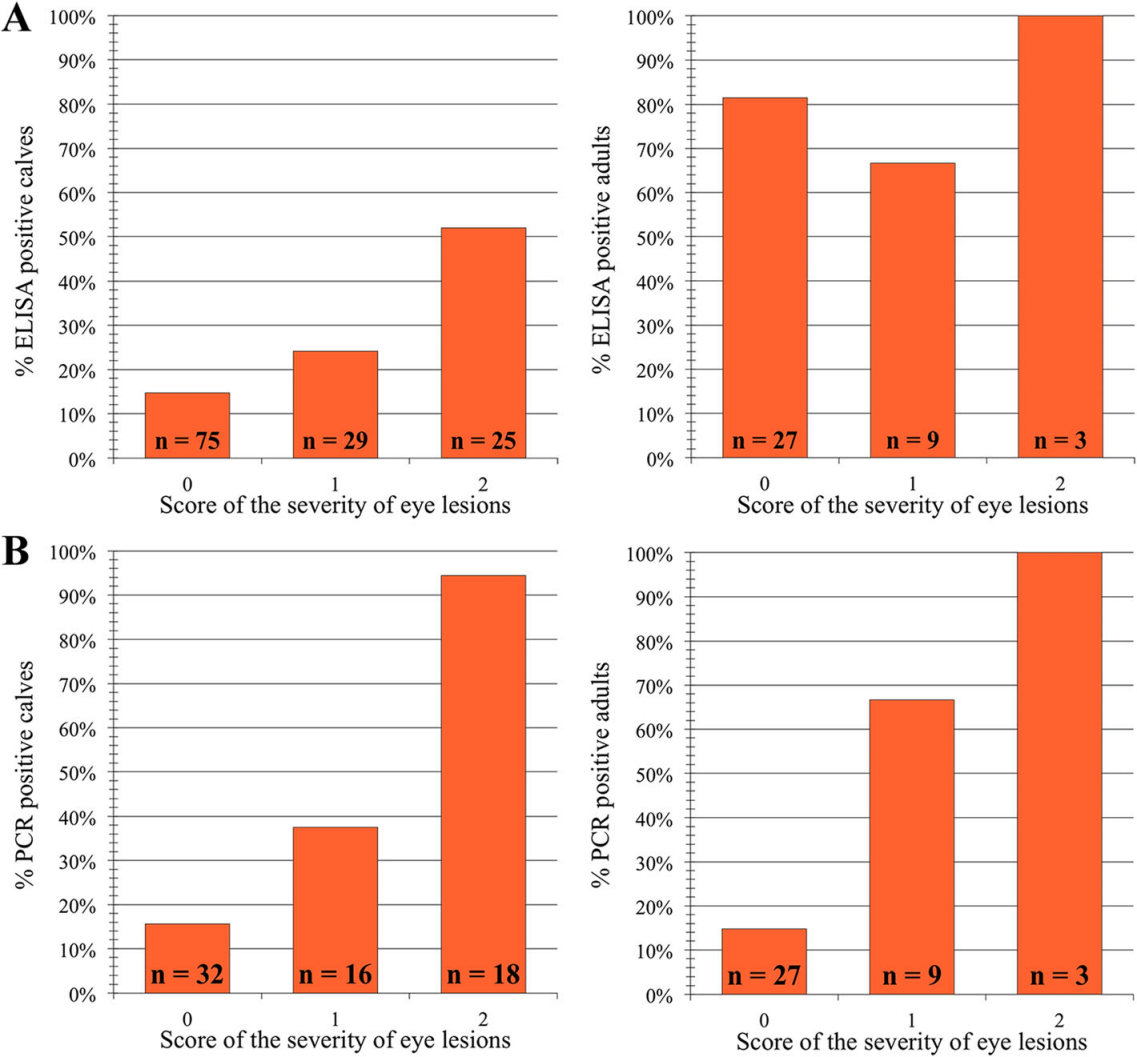
Alphaherpesvirus seroprevalence (**a**) and alphaherpesvirus PCR results (eye swab) (**b**) among reindeer without eye lesions (0), with mild (1), or moderate/severe (2) eye lesions presented as percentage comparing calves (left) and adults (right)

The CvHV2 PCR results showed a positive significant association between the presence of CvHV2 DNA in the conjunctival swab samples and the severity of the clinical signs of IKC. This association was valid both for the total number of animals independently of age-class (*p* < 0.001) and also for calves (*p* = 0.002) and adults (*p* = 0.022) as separate groups (Figs. 3 & 4). Nine out of 59 of the asymptomatic animals (15.3%) had CvHV2 DNA in their eyes.

### *Chlamydiacea* detection

DNA specific for *Chlamydiacea* was detected in 16 of 135 animals (11.9%), eight with and eight without clinical signs of IKC. No statistical association could be established (*p* = 1.000) between the presence of clinical signs of IKC and the detection of bacteria from the family *Chlamydiacea*.

### *Mycoplasmaconjunctivae* detection

DNA specific for *M. conjunctivae* was detected in 2 of 197 individuals (1.0%). No link between the presence of clinical signs and *M. conjunctivae* could be established (*p* = 1.000).

### Bacteriological investigations

A total of 202 animals, one eye from each, were sampled for bacteriology. Bacteria were cultivated from the conjunctiva of 152 reindeer, of which 35 presented clinical signs of IKC, 52 did not show signs of IKC, and the clinical status was unknown for 65 individuals. In animals from which clinical information was not available, bacteria such as *Acinetobacter* spp. (*n* = 13), *Aeromonas hydrophila* (*n* = 5), *Escherichia coli* (*n* = 9), *Micrococcus lylae* (*n* = 8) and *Staphylococcus* spp. (*n* = 3) were the major findings.

Bacteria from the genus *Moraxella* were isolated from 11 of the 51 animals showing signs of IKC (21.6%) and from 5 out of 84 with no signs (5.6%). Seven of the 11 isolates were identified at species level as *M. bovoculi*.

One of the *M. bovoculi* isolates was cultured from the eye swab from one reindeer with mild signs of IKC in Ifjordfjellet (Finmmark County, Norway) (Fig. 1). All other *Moraxella* spp. isolates were obtained from two herds with active IKC outbreaks. However, no significant differences were found between the presence of *Moraxella* spp*.* in reindeer with IKC and reindeer without IKC (*p* = 0.148).

**Fig. 5:**
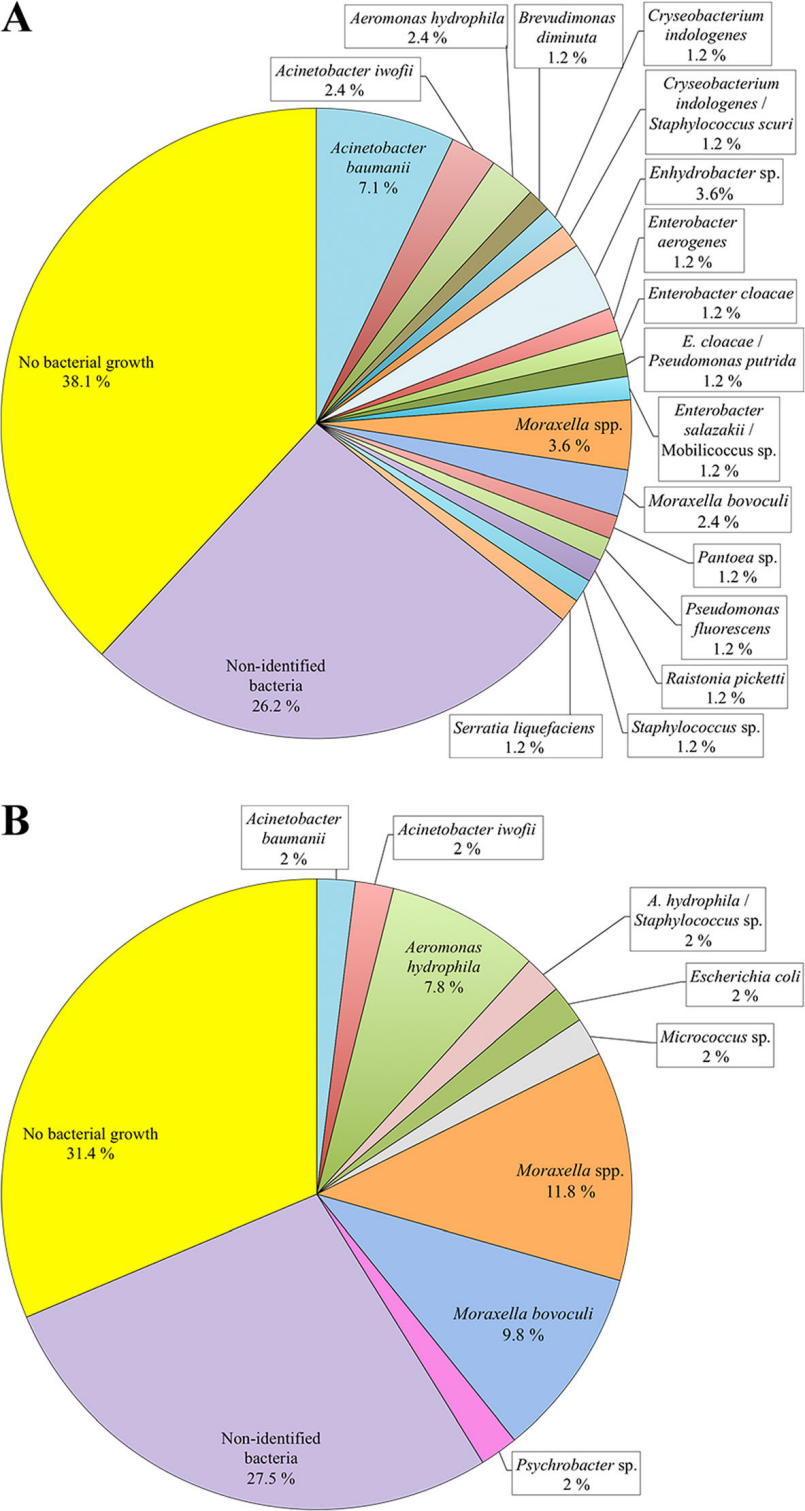
Bacterial species that dominated the culture plates inoculated with swab samples from eyes of semi-domesticated Eurasian reindeer (*Rangifer tarandus tarandus*) without (**a**) and with (**b**) clinical signs of infectious keratoconjunctivitis (IKC), including the percentage of swabs from which the bacteria were isolated

Several species of bacteria were isolated from the eyes of symptomatic and non-symptomatic animals (Fig. 5), but statistical association between the presence of any of these bacteria and clinical signs of IKC could not be established. For 52 of the reindeer (25.7%, one conjunctival swab from each animal obtained for bacteriology), the cultivation results were not conclusive due to poor bacterial growth or the absence of a dominant species, i.e. nonspecific mixed culture.

## Discussion

There was a positive correlation between the presence of antibodies against alphaherpesvirus and the presence of clinical signs of IKC in calves (*p* < 0.001), but not in adults (*p* = 0.417). Calves had higher scores of IKC clinical signs and seemed to be suffering more acute forms of the disease as compared to older animals, most likely due to the lack of previous exposures to the virus and therefore absence of immunity during the establishment of the infection. The higher seroprevalence of antibodies against alphaherpesvirus in adults as compared to calves was expected, since alphaherpesviruses produce lifelong infections [45], which is also in line with previous findings [46].

Adult animals that were previously exposed and infected could be carrying a latent infection without showing clinical signs at the time of sampling. Reactivation from this latency stage will stimulate a quick onset of the immune response protecting the animals from developing disease.

The detection of CvHV2 DNA in eye swabs by PCR and the prevalence of PCR positive individuals, which increased with the severity of the clinical signs of IKC (Fig. 3), supports the hypothesis that CvHV2 is a primary agent in the development of IKC in reindeer [15]. CvHV2 DNA was also amplified by PCR from the eyes of 15.3% of the apparently healthy reindeer. These asymptomatic animals may have been sampled during the incubation period of the disease or the virus may have been replicating in the conjunctival mucosa without producing clinical IKC signs.

It was not possible to detect CvHV2 DNA from 12 animals with mild and one with severe signs of IKC. The signs included in the first category are defined as nonspecific signs associated to IKC, such as increased lacrimation and mild conjunctivitis, which can have a non-infectious cause such as dust, dirt, allergies or minor trauma. In a natural outbreak of IKC in reindeer, the virus was most often detected and isolated at higher titers, from animals with mild or early stage signs of IKC and only from a few animals with severe signs. It was hypothesized that the peak of viral replication and shedding occurred at earlier stages, whereas secondary bacterial infections were dominant in later stages of the disease [15, 21]. It is also possible that the virus, during the late stages of IKC, is entering the latency stage, shutting down the active replication cycle and the shedding of progeny virus.

The statistical analysis of our data supports these observations. There was a strong association between the presence of CvHV2 DNA in the eye of the animals and the presence of clinical signs (*p* < 0.001). These results indicate that during an acute CvHV2 infection, with active replication of the virus in the conjunctival mucosa, clinical signs characteristic of IKC may develop.

Interestingly, all CvHV2 seropositive calves were also positive for the presence of CvHV2 DNA in their eyes (12/12). The ratio of PCR-positive animals also positive for the presence of antibodies against CvHV2 increased with the development of more severe clinical signs of IKC (Score 0 = 0% (0/5), Score 1 = 16,7% (1/6), Score 2 = 64,7% (11/17)). Those results support the hypothesis of CvHV2 infected calves seroconverting in later stages of the disease. Seronegative calves with positive PCR results (*n* = 16) might still be in the window period of the disease, in which the animals have been infected, but it is not possible to detect antibodies.

Despite serological screenings reporting antibodies against both pestivirus and gammaherpesvirus in reindeer in Fennoscandia [22, 27], little information is available about their pathogenic potential related to eye disease. The lack of association between the presence of antibodies against pestivirus and gammaherpesvirus and the presence of clinical signs of IKC indicated that these viruses do not contribute to the development of IKC in reindeer. However, it has been speculated that there might be an interaction between pestivirus and alphaherpesvirus in reindeer, in the way that infection with one of the viruses increases the chances of infection by the second virus [29]. The statistical association between the presence of antibodies against pestivirus and alphaherpesvirus in our study could reinforce this hypothesis (*p* = 0.003). Interestingly, all animals that were seropositive to both alphaherpes- and pestivirus (*n* = 10) were sampled from the same herd and period, which could also means that the animals in that herd were managed differently, increasing the possibility of exposure to the pestivirus.

Bacteria belonging to the genus *Moraxella* were isolated from the eyes of eleven animals with, and five animals without clinical signs of IKC. Seven of these isolates were characterized as *M. bovoculi*. Together with *M. bovis*, *M. bovoculi* has been identified as a causative agent of IBK in cattle [47, 48], but a randomized blinded challenge study suggested that *M. bovoculi* was not causally related with IBK in this species [49].

A statistically significant association did not exist between the isolation of *Moraxella* spp*.* and the presence clinical signs of IKC in reindeer (*p* = 0.461). However, fifteen of the samples from which *Moraxella* spp. were cultivated were obtained during two different IKC outbreaks, in Sørreisa (Norway) and Karesuando (Sweden), supporting the hypothesis of a biological significance of *Moraxella* spp. in the development of IKC in reindeer.

Dickey et al. [50] showed that there are large genomic differences between *Moraxella bovoculi* isolates from IBK affected cattle and asymptomatic animals. These results open the possibility to the presence of different strains of *Moraxella bovoculi* with different pathogenic potential. Therefore, further genomic investigations should be carried out in order to identify if pathogenic characteristics are present in the *Moraxella* spp. isolated from the eyes of apparently healthy and diseased semi-domesticated reindeer.

DNA specific for *Chlamydiacea* and *M. conjunctivae*, both known to be involved in IKC and eye infections in other host species, was detected in a few reindeer samples, both from animals with or without clinical signs of IKC. However, the lack of association between the presence of *Chlamydiacea* or *M. conjunctivae* DNA and the presence of clinical signs of IKC may suggest that these bacteria are not essential to the pathogenesis of IKC in reindeer.

A great variety of other bacteria were isolated from the eyes of both healthy and IKC affected animals (Fig. 5), including some potentially pathogenic bacteria (e.g. *Pseudomonas* spp*.* or *Staphylococcus* spp. [51, 52]), but also several bacterial species that do not have any known importance in veterinary medicine, most likely representing contamination from the environment (i.e. dust, soil, feces etc.). However, no significant association could be identified between the presence of any of the isolated bacteria and the presence of clinical signs of IKC, which may suggest that none of these bacteria have a significant importance for the development of IKC in reindeer. Bacteria did not grow in 24.8% of the cultured plates, a percentage that seems higher than those reported in other ruminant species [13, 14]. This increment could be attributed to the somewhat long (2–9 days) transport time for some samples to the laboratory which may in some cases have reduced the survival of bacteria in the samples prior to cultivation, or simply reflect a different environment (i.e. free ranging animals as opposed to livestock).

Some species of bacteria might require the presence of damage of the mucosal membrane to be able to establish an infection, as occurs with the cytopathic effect (CPE) produced by CvHV2 during its lytic cycle. A depression of the cell-mediated immunity due to virus-induced lymphocytolysis after infection with BoHV1 has been described [53], and it is relevant to think that CvHV2, a close relative to BoHV1, could produce a similar effect on the reindeer’s immune system, favoring the establishment of secondary bacterial infections. On the other hand, the possible role of bacterial infection as a trigger for the reactivation of latent CvHV2 cannot be discarded either. Bovine Herpesvirus 4 (BoHV4) can enter a lytic replication cycle from latency after endometrial infection with *E. coli*, causing uterine disease in cattle [54]. However, BoHV4 belongs to the subfamily *Gammaherpesvirinae*, and whether this finding can be applied to CvHV2 or any other ruminant alphaherpesviruses remains unclear and should be studied in further detail.

## Conclusions

Among the microorganism identified in this study, CvHV2 is the most plausible candidate as the causative agent of IKC in semi-domesticated reindeer, and pestivirus and gammaherpesvirus may be discarded as primary causative agents of IKC in reindeer. The isolation of *M. bovoculi* during two different outbreaks of IKC makes this bacterial species an alternative candidate as a possible primary agent of this disease, or maybe more likely, as a secondary and opportunistic pathogen, following a CvHV2 infection as is probably the case for other bacterial species identified in this study. Further studies should be carried out to better understand the infection biology and the pathogenesis of IKC in reindeer.

## Abbreviations

16S rRNA: 16S ribosomal RNA
BDV: Border disease virus
bELISA: Blocking enzyme-linked immunosorbent assay
BoHV1: Bovine Herpesvirus 1
BoHV4: Bovine Herpesvirus 4
BVDV: Bovine viral diarrhea virus
cELISA: Competitive enzyme-linked immunosorbent assay
CPE: Cytopathic effect
C_t_: Threshold cycle
CvHV2: Cervid Herpesvirus 2
DEPC: Diethylpryocarbonate
EMEM: Eagle’s Minimum Essential Medium
gB: Glycoprotein B
IBK: Infectious bovine keratoconjunctivitis
IKC: Infectious keratoconjunctivitis
MCF: Malignant catarrhal fever
MCFV: Malignant catarrhal fever virus
PCR: Polymerase chain reaction
